# Characteristics of gastric precancerous conditions and *Helicobacter pylori* infection among dyspeptic patients in north-eastern Iran: is endoscopic biopsy and histopathological assessment necessary?

**DOI:** 10.1186/s12885-021-08626-6

**Published:** 2021-10-26

**Authors:** Abbas Esmaeilzadeh, Ladan Goshayeshi, Robert Bergquist, Lida Jarahi, Alireza Khooei, Alireza Fazeli, Hooman Mosannen Mozaffari, Ali Bahari, Mohammad Bagher Oghazian, Benyamin Hoseini

**Affiliations:** 1 Department of Gastroenterology and Hepatology, Faculty of Medicine, Mashhad University of Medical Sciences, Mashhad, Iran; 2 Surgical Oncology Research Center, Mashhad University of Medical Sciences, Mashhad, Iran; 3 Ingerod, SE-454 94, Brastad, Sweden; 4 Formerly UNICEF/UNDP/World Bank/WHO Special Programme for Research and Training in Tropical Diseases (TDR), World Health Organization, Geneva, Switzerland; 5 Community Medicine Department, Faculty of Medicine, Mashhad University of Medical sciences, Mashhad, Iran; 6 Department of Pathology, Faculty of Medicine, Mashhad University of Medical Sciences, Mashhad, Iran; 7 Cardiology Resident, Faculty of Medicine, Mashhad University of Medical Sciences, Mashhad, Iran; 8 Department of Internal Medicine, Faculty of Medicine, North Khorasan University of Medical Sciences, Bojnurd, Iran; 9 Pharmaceutical Research Center, Mashhad University of Medical Sciences, Mashhad, Iran; 10 Department of Health Information Technology, Neyshabur University of Medical Sciences, Neyshabur, Iran

**Keywords:** Gastric biopsy, Histopathology, *Helicobacter pylori*, Precancerous conditions, Addiction, Iran

## Abstract

**Background:**

Early detection and appropriate treatment of precancerous, mucosal changes could significantly decrease the prevalence of life-threatening gastric cancer. Biopsy of the normal-appearing mucosa to detect *Helicobacter pylori* and these conditions is not routinely obtained. This study assesses the prevalence and characteristics of *H. pylori* infection and precancerous conditions in a group of patients suffering from chronic dyspepsia who were subjected to gastric endoscopy and biopsy mapping.

**Methods:**

This cross-sectional study included dyspeptic patients, not previously treated for *H. pylori*, undergoing esophagogastroduodenoscopy (EGD) with their gastric endoscopic biopsies obtained for examination for evidence of *H. pylori* infection and precancerous conditions. Demographic and clinical data on the gender, smoking, opium addiction, alcohol consumption, medication with aspirin, corticosteroids and non-steroidal anti-inflammatory drugs (NSAIDs) and family history of cancer were collected by interviewing the patients and evaluating their health records. The cohort examined consisted of 585 patients with a mean (SD) age of 48.0 (14.46) years, 397 (67.9%) of whom were women.

**Results:**

*H. pylori* infection was identified in 469 patients (80.2%) with the highest prevalence (84.2%) in those aged 40–60 years. Opium addiction correlated with a higher a *H. pylori* infection rate, while alcohol consumption was associated with a lower rate by Odds Ratio 1.98 (95% CI 1.11–3.52) and 0.49 (95% CI 0.26–0.92), respectively. The prevalence of intestinal metaplasia, gastric atrophy and gastric dysplasia was 15.2, 12.6 and 7.9%, respectively. Increased age, positive *H. pylori* infection, endoscopic abnormal findings and opium addiction showed a statistically significant association with all precancerous conditions, while NSAID consumption was negatively associated with precancerous conditions. For 121 patients (20.7% of all), the EGD examination revealed normal gastric mucosa, however, for more than half (68/121, 56.2%) of these patients, the histological evaluation showed *H. pylori* infection, and also signs of atrophic mucosa, intestinal metaplasia and dysplasia in 1.7, 4.1 and 1.7%, respectively.

**Conclusion:**

EGD with gastric biopsy mapping should be performed even in the presence of normal-appearing mucosa, especially in dyspeptic patients older than 40 years with opium addiction in north-eastern Iran. Owing to the high prevalence of precancerous conditions and *H. pylori* infection among patients with dyspepsia in parts of Iran, large-scale national screening in this country should be beneficial.

## Introduction

Gastric cancer is still a major worldwide problem, ranking fifth for incidence and third for cancer-related mortality [1–3]. According to GLOBOCAN 2018, gastric carcinoma is the second most common cancer in Iran and the 5-year prevalence is 19.22 per 100,000 [2]. Chronic atrophic gastritis and intestinal metaplasia are considered precancerous conditions and they constitute the background, against which dysplasia and more serious histological changes may occur [4]. Intestinal-type gastric adenocarcinoma represents the final outcome of the inflammation–atrophy–metaplasia–dysplasia–carcinoma sequence [5]. Even though early recognition and treatment is possible, most cases are diagnosed at a late stage and thus a large number of patients diagnosed with gastric cancer die of the disease [1]. Early detection and treatment, primarily by endoscopy rather than invasive surgery, is recommended [6, 7].

Multiple risk factors have been linked to the multistep progression from precancerous conditions to gastric cancer [8–10]. *Helicobacter pylori* infection plays a pivotal role in this progression, and a recent meta-analysis indicates that testing and treating this infection when found is associated with a reduced incidence of gastric cancer [11, 12]. This indicates the importance of knowing the distribution and prevalence of *H. pylori* infections and precancerous conditions and related risk factors, including the strategies suitable for lowering the incidence of gastric cancer. Although this would clearly help to prevent gastric cancer, few studies on the incidence of gastric precancerous conditions and *H. pylori* infection have been published in Iran. However, Ajdarkosh et al. [13] have found prevalence of *H. pylori* infection, intestinal metaplasia, gastric atrophy and dysplasia in 64.5, 19.8, 12.8 and 3.2%, respectively, among chronically dyspeptic patients aged ≥40 years. They recommend upper endoscopy and gastric mapping sampling in intermediate-risk to high-risk areas. Another study in Ardabil province [14], which has a high rate of gastric cancer, found that atrophic gastritis, reactive atypia and intestinal metaplasia are common in the antrum, corpus and cardia of the stomach and they therefore recommend endoscopic screening for precancerous conditions.

Esophagogastroduodenoscopy (EGD) is commonly performed when evaluating patients with dyspepsia [15]. In many cases with normal-appearing mucosa, *H. pylori* and precancerous conditions can still be present in the stomach, and a reliable diagnosis is therefore important. In the absence of endoscopically visible lesions, biopsies from the stomach would contribute to diagnosing most *H. pylori*-related inflammatory and precancerous gastric lesions, which would be misdiagnosed without access to biopsies [16].

The American Gastroenterological Association (AGA) currently recommends biopsy even from normal appearing mucosa for the detection of *H. pylori* infection if previously unknown in the patient [15, 17]. However, to our knowledge, there are no clinical standards or guidelines for the performance of biopsies with respect to *H. pylori* and precancerous conditions of normal-appearing gastric mucosa in Iranian patients. The additional expense incurred by the need for obtaining and interpreting more biopsies from normal mucosa [18] may be cancelled out by the lower numbers of people developing gastric cancer. Early diagnosis of precancerous conditions and *H. pylori* infection would no doubt lead to an effective approach which would allow the early detection of gastric cancer, especially in high-risk areas like Iran. To that end, this study would assessed the prevalence and characteristics of *H. pylori* infection and precancerous conditions in patients with normal and abnormal-appearing mucosa undergoing EGD with dyspepsia as the sole indication.

## Material and methods

### Study summary

**Fig. 1:**
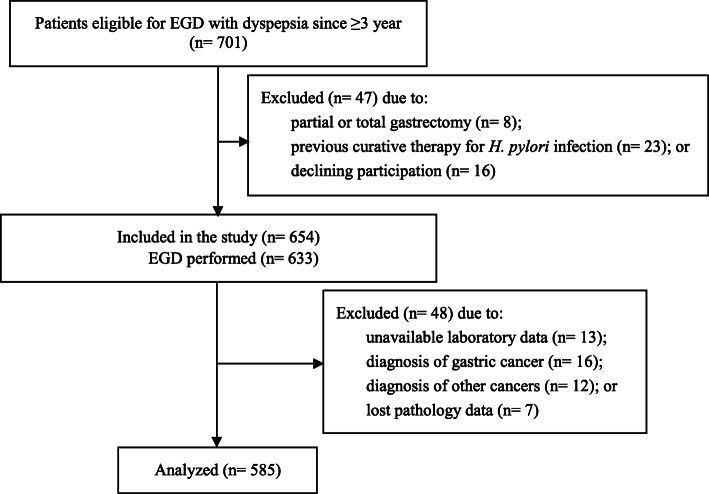
Flow diagram of patient recruitment

We conducted a cross-sectional study from 2015 to 2017 based on clinical data, bacterial findings and histological classification of the gastric mucosa in a set of patients with dyspepsia at the Gastroenterology Clinic of Emam Reza Hospital, a tertiary referral hospital affiliated with Mashhad University of Medical Sciences (MUMS). The inclusion/exclusion process was done as shown in Fig. 1.

### Participants

During the three study years, patients aged > 18 years with dyspepsia, consistent with the Rome III criteria [19], which include one or more of postprandial fullness, early satiation, epigastric pain and/or burning, and thus eligible for EGD (*n* = 701), were enrolled. Patients were excluded if they had any history of partial/total gastrectomy or previous therapy for *H. pylori* infection. At this point, 16 patients opted out of the study for unknown reasons leaving 654 for the next step. However, EGD was only performed on 633 patients since 21 were unwilling to undergo the examination. A further 48 patients were excluded for various reasons (Fig. 1) resulting in 585 participants in the study.

### Data gathering

Demographic data and clinical characteristics, including data on age, gender, past medical history (diabetes and hypertension in particular), tobacco use (daily smoking), alcohol use (daily drinker), opium addiction (daily user > 6 months), taking ≥500 mg of aspirin for > 1 month or other non-steroidal anti-inflammatory drugs (NSAIDs), family history of cancer (especially in first-degree relatives (FDRs) and/or second-degree relatives (SDRs) were collected by interviewing the patients and evaluating their health records before the EGD step.

### Histopathology

Endoscopic biopsies were fixed in adequately buffered formalin (10%) overnight and subsequently routinely processed. All fragments were embedded in one paraffin block, multiple serial sections were obtained and all stained by routine hematoxylin/eosin and Giemsa stains. The slides were examined by two pathologists and in case of any diagnostic disagreement, other colleagues were asked to review the slides. Furthermore, macro-endoscopic features observed during the gastric inspection were also reviewed.

Precancerous conditions refer to a variety of conditions in which changes in stomach cells make them more prone into cancer. In our study, these included mucosal atrophy, intestinal metaplasia, dysplasia and mucosa with abnormal gastric biopsy. Gastric atrophy is considered if there are loss of gastric glands, either together with pure (non-metaplastic) or associated with pyloric or intestinal metaplasia (metaplastic type). The degree of atrophy is graded as mild, moderate or severe; however, in this study, all types of atrophy was is considered regardless of severity. Gastric dysplasia, regarded as a combination of architectural atypia (glandular crowding, budding and branching) and cytologic atypia (cellular overlapping, hyperchromasia of the nuclei, pseudostratification, pleomorphism, dispolarity, increased mitosis and lack of surface maturation), was classified as adenomatous (intestinal) or foveolar (gastric). Regarding the extent of these changes, dysplasia was graded as low or high; however, all types of dysplasia were considered regardless of severity.

### Expected outcomes

The primary focus was on finding precancerous conditions and *H. pylori* infection. A secondary focus was to evaluate potential associations between the histopathological examinations and the responses collected.

### Statistical analysis

The chi square test was used to determine differences in the categorical variables. The Shapiro–Wilk test was used to examine the degree of data normality, while T-test, ANOVA, Mann–Whitney or Kruskal–Wallis tests were used to investigate the normality distribution of the data, i.e. to identify potential, significant differences between independent variables. Multivariable logistic regression analysis with backward stepwise model was used to ascertain the statistical significance of the association between precancerous conditions on the one hand and demographic data, clinical characteristics and *H. pylori* presence on the other. A two-tailed *p-*value of less than 0.05 was considered statistically significant. The analyses were performed using SPSS statistics (version 20.0, IBM Corp., Armonk, NY, USA).

## Results

Demographic data, clinical characteristics including *H. pylori* status and histological patterns from the 585 patients (67.9% women) were analyzed (Table 1 and Table 2). The mean (±SD) age of the participants was 48.0 (±14.46) years. Also, 146 (25.0%) patients were addicted to opium or methamphetamine. Alcohol consumption was reported in 50 (8.5%) of the patients, while 173 (29.6%) regularly used NSAIDs and 88 (15.0%) aspirin. Histologic findings in endoscopic biopsies from the patients revealed that the prevalence of intestinal metaplasia, gastric atrophy and gastric dysplasia was 15.2, 12.6, and 7.9%, respectively. *H. pylori* infection rates were found in as many as 80.2% of the patients, with a particularly high prevalence among those aged 40–60 years. Opium addiction indicated higher *H. pylori* infection rates, while the situation was the opposite with respect to alcohol consumption (OR 1.98, %95 CI 1.11–3.52 and OR 0.49, 95% CI 0.26–0.92, respectively).

**Table 1:** Basic characteristics of the study participants

Common risk factors	Metaplasia	Atrophy	Dysplasia	Abnormal gastric biopsy	No pathological finding	Total
Age (years)						
≤ = 40	5 (2.3)	2 (1.0)	2 (1.0)	59 (27.4)	147 (68.3)	215
41–50	19 (15.3)	13 (10.5)	16 (12.9)	16 (12.9)	60 (48.4)	124
51–60	24 (19.5)	22 (17.9)	9 (7.3)	16 (13.0)	52 (42.3)	123
≥ = 61	41 (33.3)	37 (30.1)	19 (15.4)	12 (9.7)	14 (11.4)	123
Sex						
Female	52 (13.1)	41 (10.3)	32 (8.1)	78 (19.6)	194 (48.9)	397
Male	37 (19.7)	33 (17.5)	14 (7.4)	25 (13.3)	79 (42.0)	188
*H. pylori* infection	83 (17.7)	68 (14.5)	42 (8.9)	52 (11.1)	224 (47.8)	469
Smoking	20 (17.8)	17 (15.2)	9 (8.0)	16 (14.3)	50 (44.6)	112
NSAID use	13 (7.5)	11 (6.3)	6 (3.5)	27 (15.6)	116 (67.0)	173
Aspirin	8 (9.1)	13 (14.8)	6 (6.8)	15 (17.0)	46 (52.3)	88
Addiction (opium and/or methamphetamine)	35 (24.0)	30 (20.5)	17 (11.6)	19 (13.0)	45 (30.8)	146
Alcohol consumption	6 (12.0)	6 (12.0)	3 (6.0)	8 (16.0)	27 (54.0)	50

NSAID, nonsteroidal anti-inflammatory drug

**Table 2:** Association of precancerous conditions, abnormal gastric biopsy, atrophy, metaplasia and dysplasia with demographic and clinical characteristics of the dyspeptic patients

Categorical variable	Precancerous conditions (***n*** = 585)		***P***-value	Abnormal gastric biopsy (***n*** = 585)		***P***-value	Atrophy (***n*** = 585)		***P***-value	Metaplasia (***n*** = 585)		***P***-value	Dysplasia (***n*** = 585)		***P***-value
	Yes (***n*** = 122)	No (***n*** = 463)		Yes (***n*** = 103)	No (***n*** = 482)		Yes (***n*** = 74)	No (***n*** = 511)		Yes (***n*** = 89)	No (***n*** = 496)		Yes (***n*** = 46)	No (***n*** = 539)	
	n (%)	n (%)		n (%)	n (%)		n (%)	n (%)		n (%)	n (%)		n (%)	n (%)	
Age (years)															
< = 40	7 (5.7)	208 (44.9)	**0.001**	59 (57.3)	156 (32.4)	**0.001**	2 (2.7)	213 (41.7)	**0.001**	5 (5.6)	210 (42.3)	**0.001**	2 (4.3)	213 (39.5)	**0.001**
41–50	33 (27.1)	91 (19.7)		16 (15.5)	107 (22.4)		13 (17.6)	111 (21.7)		19 (21.3)	105 (21.2)		16 (34.8)	108 (20.0)	
51–60	34 (27.9)	89 (19.2)		16 (15.5)	108 (22.2)		22 (29.7)	101 (19.8)		24 (27.0)	99 (20.0)		9 (19.6)	114 (21.2)	
> = 61	48 (39.3)	75 (16.2)		12 (11.7)	111 (23.0)		37 (50.0)	86 (16.8)		41 (46.1)	82 (16.5)		19 (41.3)	104 (19.3)	
Sex															
Female (*n* = 397)	76 (62.3)	321 (69.3)	0.15	78 (75.7)	319 (66.2)	0.06	41 (55.4)	356 (69.7)	**0.01**	52 (58.4)	345 (69.6)	**0.04**	32 (69.6)	365 (67.7)	0.79
Male (*n* = 188)	46 (37.7)	142 (30.7)		25 (24.3)	163 (33.8)		33 (44.6)	155 (30.3)		37 (41.6)	151 (30.4)		14 (30.4)	174 (32.3)	
Smoking															
Yes (*n* = 112)	25 (20.5)	87 (18.8)	0.69	16 (15.5)	96 (19.9)	0.38	17 (23.0)	95 (18.6)	0.37	20 (22.5)	92 (18.5)	0.39	9 (19.6)	103 (19.1)	0.94
No (*n* = 473)	97 (79.5)	376 (81.2)		87 (84.5)	386 (80.1)		57 (77.0)	416 (81.4)							
										69 (77.5)	404 (81.5)		37 (80.4)	436 (80.9)	
Addiction^*^															
Yes (*n* = 146)	43 (35.2)	103 (22.2)	**0.003**	19 (18.4)	127 (26.3)	0.09	30 (40.5)	116 (22.7)	**0.001**	35 (39.3)	111 (22.4)	**0.001**	17 (36.9)	129 (23.9)	**0.05**
No (*n* = 439)	79 (64.8)	360 (77.8)		84 (81.6)	355 (73.7)		44 (59.5)	395 (77.3)		54 (60.7)	385 (77.6)		29 (63.1)	410 (76.1)	
Alcohol consumption															
Yes (*n* = 50)	11 (9.0)	39 (8.4)	0.85	8 (7.8)	42 (8.7)	0.85	6 (8.1)	44 (8.6)	0.88	6 (6.7)	44 (8.9)	0.86	3 (6.5)	47 (8.7)	0.78
No (*n* = 535)	111 (81.0)	424 (91.6)		95 (92.2)	440 (91.3)		68 (91.9)	467 (91.4)		83 (93.3)	452 (91.1)		43 (93.5)	492 (91.3)	
NSAID use															
Yes (*n* = 173)	18 (14.7)	155 (33.5)	**0.001**	27 (26.2)	146 (30.3)	0.47	11 (14.9)	162 (31.7)	**0.003**	13 (14.6)	160 (32.3)	**0.001**	6 (13.0)	167 (31.0)	**0.01**
No (*n* = 412)	104 (85.3)	308 (66.5)		76 (73.8)	336 (69.7)		63 (85.1)	349 (68.3)		76 (85.4)	336 (67.7)		46 (87.0)	372 (69.0)	
Aspirin use															
Yes (*n* = 88)	15 (12.3)	73 (15.8)	0.39	15 (14.6)	73 (15.1)	1.0	13 (17.6)	75 (14.7)	0.51	8 (9.0)	80 (16.1)	0.08	6 (13.0)	82 (15.2)	0.69
No (*n* = 497)	107 (87.7)	390 (84.2)		88 (85.4)	409 (84.9)		61 (82.4)	436 (85.3)		81 (91.0)	416 (83.9)		40 (87.0)	457 (84.8)	
Diabetes (types I and II)															
Yes (*n* = 99)	20 (16.4)	79 (17.1)	1.0	13 (12.6)	86 (17.8)	0.25	15 (20.3)	84 (16.4)	0.41	18 (20.2)	81 (16.3)	0.37	6 (13.0)	93 (17.3)	0.46
No (*n* = 486)	102 (83.6)	384 (82.9)		90 (87.4)	396 (82.2)		59 (79.7)	427 (83.6)		71 (79.8)	415 (83.7)		40 (87.0)	446 (82.7)	
*H. pylori* infection															
Yes (*n* = 469)	114 (93.4)	355 (76.7)	**0.001**	52 (50.5)	417 (86.5)	**0.001**	68 (91.9)	401 (78.5)	**0.007**	83 (93.3)	383 (77.8)	**0.001**	42 (91.3)	427 (79.2)	**0.048**
No (*n* = 116)	8 (6.6)	108 (23.3)		51 (49.5)	65 (13.5)		6 (8.1)	110 (21.5)		6 (6.7)	110 (22.2)		4 (8.7)	112 (20.8)	
Dyspepsia pattern															
EPS (*n* = 245)	50 (41.0)	195 (42.1)	0.99	46 (44.7)	199 (41.3)	0.45	31 (41.9)	214 (41.9)	0.99	37 (41.6)	208 (41.9)	0.99	17 (37.0)	228 (42.3)	0.46
Flatulence (*n* = 143)	30 (24.6)	113 (24.4)		20 (19.4)	123 (25.5)		18 (24.3)	125 (24.5)		22 (24.7)	121 (24.4)		9 (19.6)	134 (24.9)	
Early satiation (*n* = 48)	11 (9.0)	37 (8.0)		6 (5.8)	42 (8.7)		7 (9.5)	41 (8.0)		7 (7.9)	41 (8.3)		6 (13.0)	42 (7.8)	
PF (*n* = 48)	10 (8.2)	38 (8.2)		9 (8.2)	39 (8.2)		6 (8.1)	42 (8.2)		8 (9.0)	40 (8.1)		6 (13.0)	42 (7.8)	
Reflux (*n* = 101)	21 (17.2)	80 (17.3)		22 (21.4)	79 (16.4)		12 (16.2)	89 (17.4)		15 (16.9)	86 (17.3)		8 (17.4)	93 (17.3)	
Endoscopic findings															
Normal (*n* = 121)	5 (4.1)	116 (25.0)	**0.001**	5 (4.1)	116 (25.0)	**0.001**	2 (2.7)	119 (23.3)	**0.001**	5 (5.6)	116 (23.4)	**0.001**	2 (4.3)	119 (22.1)	**0.001**
Erythema (*n* = 176)	23 (18.8)	153 (33.1)		23 (18.8)	153 (33.1)		10 (13.5)	166 (32.5)		12 (13.5)	164 (33.1)		12 (26.1)	164 (30.4)	
M o/s (*n* = 151)	33 (27.1)	118 (25.5)		33 (27.1)	118 (25.5)		18 (24.3)	133 (26.0)		20 (22.5)	131 (26.4)		12 (26.1)	139 (25.8)	
Erosion (*n* = 68)	18 (14.7)	50 (10.8)		18 (14.7)	50 (10.8)		11 (14.9)	57 (11.2)		17 (19.1)	51 (10.3)		6 (13.0)	62 (11.5)	
GDU (*n* = 36)	21 (17.2)	15 (3.2)		21 (17.2)	15 (3.2)		12 (16.2)	24 (4.7)		16 (18.0)	20 (4.0)		7 (15.2)	29 (5.4)	
Atrophy (*n* = 33)	22 (18.1)	11 (2.4)		22 (18.1)	11 (2.4)		21 (28.4)	12 (2.3)		19 (21.3)	14 (2.8)		7 (15.2)	26 (4.8)	
FHGC															
None (*n* = 397)	89 (73.0)	308 (66.5)	0.77	75 (72.8)	322 (66.8)	**0.012**	50 (67.6)	347 (67.9)	0.87	61 (68.5)	336 (67.7)	0.98	38 (82.6)	359 (66.6)	0.53
GC (*n* = 69)	12 (9.9)	57 (12.3)		13 (12.6)	56 (11.6)		7 (9.5)	62 (12.1)		11 (12.4)	58 (11.7)		3 (6.5)	66 (12.2)	
IC (n = 1)	0 (0.0)	1 (0.2)		1 (1.0)	0 (0.0)		0 (0.0)	1 (0.2)		0 (0.0)	1 (0.2)		0 (0.0)	1 (0.2)	
CC (*n* = 81)	17 (13.9)	64 (13.8)		5 (4.9)	76 (15.8)		14 (18.9)	67 (13.1)		13 (14.6)	68 (13.7)		4 (8.7)	77 (14.3)	
OC (*n* = 12)	2 (1.6)	10 (2.2)		2 (1.9)	10 (2.1)		1 (1.4)	11 (2.2)		2 (2.2)	10 (2.0)		0 (0.0)	12 (2.2)	
LC (*n* = 14)	1 (0.8)	13 (2.8)		4 (3.9)	10 (2.1)		1 (1.4)	13 (2.5)		1 (1.1)	13 (2.6)		1 (2.2)	13 (2.4)	
PC (*n* = 11)	1 (0.8)	10 (2.2)		3 (2.9)	8 (1.7)		1 (1.4)	10 (2.0)		1 (1.1)	10 (2.0)		0 (0.0)	11 (2.0)	
FHNGC															
None (*n* = 504)	112 (91.8)	392 (84.7)	0.12	81 (78.6)	423 (87.8)	**0.04**	66 (89.2)	438 (85.7)	0.15	81 (91.0)	423 (85.3)	0.15	42 (91.3)	462 (85.7)	0.64
Breast (*n* = 34)	2 (1.6)	32 (6.9)		8 (7.8)	26 (5.4)		1 (1.4)	33 (6.5)		1 (1.1)	33 (6.7)		2 (4.3)	32 (5.9)	
Cervical (*n* = 6)	1 (0.8)	5 (1.1)		2 (1.9)	4 (0.8)		1 (1.4)	5 (1.0)		1 (1.1)	5 (1.0)		0 (0.0)	6 (1.1)	
Ovarian (*n* = 6)	2 (1.6)	4 (0.9)		2 (1.9)	4 (0.8)		2 (2.7)	4 (0.8)		2 (2.2)	4 (0.8)		1 (2.2)	5 (0.9)	
Bladder (*n* = 4)	1 (0.8)	3 (0.6)		2 (1.9)	2 (0.4)		1 (1.4)	3 (0.6)		1 (1.1)	3 (0.6)		0 (0.0)	4 (0.7)	
Kidney (*n* = 6)	1 (0.8)	4 (0.9)		3 (2.9)	2 (0.4)		1 (1.4)	4 (0.8)		0 (0.0)	5 (1.0)		1 (2.2)	4 (0.7)	
Haematological (*n* = 12)	0 (0.0)	12 (2.6)		2 (1.9)	10 (2.1)		0 (0.0)	12 (2.3)		0 (0.0)	12 (2.4)		0 (0.0)	12 (2.2)	
Pulmonary (*n* = 14)	3 (2.5)	11 (2.4)		3 (2.9)	11 (2.3)		2 (2.7)	12 (2.3)		3 (3.4)	11 (2.2)		0 (0.0)	14 (2.6)	
**Continuous variable**	**mean ± SD**	**mean ± SD**	***P*-value**	**mean ± SD**	**mean ± SD**	***P*-value**	**mean ± SD**	**mean ± SD**	***P*-value**	**mean ± SD**	**mean ± SD**	***P*-value**	**mean ± SD**	**mean ± SD**	***P*-value**
Age (years)	57.8 ± 13.1	45.4 ± 13.7	**0.001**	41.6 ± 12.6	49.3 ± 14.5	**0.001**	61.0 ± 12.6	46.1 ± 13.7	**0.001**	59.4 ± 13.2	45.9 ± 13.7	**0.001**	58.6 ± 14.3	47.1 ± 14.1	**0.001**
Haemoglobin (Hgb)	13.3 ± 1.6	13.3 ± 1.5	0.48	13.4 ± 1.5	13.3 ± 1.5	0.62	12.9 ± 1.5	13.4 ± 1.5	0.14	13.1 ± 1.6	13.4 ± 1.5	0.12	13.4 ± 1.6	13.3 ± 1.5	0.48
25(OH)D_3_ vitamin	21.7 ± 13.0	18.1 ± 11.2	**0.002**	18.0 ± 11.5	19.0 ± 11.7	0.27	20.8 ± 12.4	18.5 ± 11.6	0.14	22.3 ± 13.7	18.2 ± 11.2	**0.004**	21.9 ± 11.2	18.6 ± 11.7	**0.013**

*EPS* Epigastric pain syndrome; *M o/s* Mucosal oedema/swelling; *PF* Postprandial fullness; *GDU* Gastric or duodenal ulcer; *FHGC* Family history of gastro-intestinal cancer; *FHNGC* Family history of non-gastro/intestinal cancer; *GC* gastric cancer; *IC* Intestinal cancer; *CC* Colon cancer; *OC* Oesophagus cancer; *LC* Liver cancer; *PC* Pancreas cancer. ^*^Addiction = opium and/or methamphetamine

Based on multivariable logistic regression analysis, increased age and positive *H. pylori* infection indicated a significant association with precancerous conditions. In contrast, NSAID consumption showed a negative association with investigated outcomes (Table 3).

**Table 3:** Association between precancerous conditions and variables investigated

Type of pathology	Variation	Univariate logistic regression		Multivariate logistic regression	
		OR (95% CI)	***P***-value	OR (95% CI)	***P***-value
Precancerous conditions	Age ≤ 40 years	Reference	–	Reference	–
	Age between 41 and 50 years	10.77 (4.59–25.26)	**0.001**	10.78 (4.54–25.59)	**0.001**
	Age between 51 and 60 years	11.35 (4.84–26.57)	**0.001**	9.88 (4.18–23.35)	**0.001**
	Age ≥ 61 years	19.01 (8.24–43.86)	**0.001**	18.53 (7.94–43.26)	**0.001**
	NSAID consumption	0.34 (0.20–0.58)	**0.001**	0.34 (0.19–0.60)	**0.001**
	*H. pylori* infection	4.33 (2.05–9.16)	**0.001**	4.01 (1.83–8.78)	**0.001**
Abnormal gastric biopsy	Age between 41 and 50 years	2.55 (1.39–4.67)	**0.002**	2.32 (1.22–4.39)	**0.01**
	Age between 51 and 60 years	2.52 (1.38–4.63)	**0.003**	2.16 (1.14–4.08)	**0.01**
	Age ≥ 61 years	3.49 (1.79–6.81)	**0.001**	3.28 (1.63–6.60)	**0.001**
	*H. pylori* infection	6.29 (3.94–10.01)	**0.001**	5.92 (3.67–9.55)	**0.001**
Atrophy	Age between 41 and 50 years	12.47 (2.76–56.25)	**0.001**	11.96 (2.64–52.20)	**0.001**
	Age between 51 and 60 years	23.19 (5.35–100.56)	**0.001**	20.19 (4.64–87.90)	**0.001**
	Age ≥ 61 years	45.82 (10.80–194.30)	**0.001**	43.29 (10.16–184.33)	**0.001**
	NSAID consumption	0.37 (0.19–0.73)	**0.004**	0.40 (0.20–0.82)	**0.012**
	*H. pylori* infection	3.1 (1.31–7.35)	**0.01**	2.72 (1.10–6.71)	**0.029**
Metaplasia	Age between 41 and 50 years	7.6 (2.76–20.92)	**0.001**	7.31 (2.63–20.29)	**0.001**
	Age between 51 and 60 years	10.18 (3.77–27.47)	**0.001**	8.75 (3.22–23.79)	**0.001**
	Age ≥ 61 years	21.00 (8.01–55.00)	**0.001**	20.00 (7.57–52.84)	**0.001**
	NSAID consumption	0.35 (0.19–0.66)	**0.001**	0.37 (0.19–0.71)	**0.003**
	*H. pylori* infection	3.94 (1.67–9.27)	**0.002**	3.58 (1.47–8.72)	**0.005**
Dysplasia	Age between 41 and 50 years	15.77 (3.56–69.87)	**0.001**	16.29 (3.66–72.38)	**0.001**
	Age between 51 and 60 years	8.40 (1.78–39.57)	**0.013**	7.65 (1.62–36.11)	**0.013**
	Age ≥ 61 years	19.45 (4.44–85.11)	**0.001**	18.64 (4.25–81.79)	**0.001**
	NSAID consumption	0.33 (0.13–0.80)	**0.014**	0.33 (0.13–0.82)	**0.017**

*OR* odds ratio

For 121 patients (20.7% of all), the EGD examination revealed normal gastric mucosa; however, for more than half (68/121–56.2%) of these patients, the histological evaluation showed *H. pylori* infection and also signs of atrophic mucosa, intestinal metaplasia and dysplasia in 1.7, 4.1 and 1.7%, respectively.

## Discussion

To our knowledge, this is the first study to assess the prevalence of advanced gastric conditions and related factors in north-eastern Iran. The study found a considerable prevalence of *H. pylori* infection, intestinal metaplasia, gastric atrophy and gastric dysplasia among the 585 study participants in the study area. Overall, our findings with respect to *H. pylori* infections and endoscopic abnormal findings and their association with older age are in line with other studies performed in Iran [20–23]. Importantly, some patients without visible endoscopic lesions turned out to have *H. pylori* infections and related precancerous conditions, which might have been missed without access to biopsies.

In our study, increased age and positive *H. pylori* infection indicated significant association with precancerous conditions. Given that dysplasia condition has been shown a high degree of progression to cancer, the association with dysplasia is more important compared to the other conditions. Consequently, it is necessary to apply endoscopic resection once high-grade dysplasia is observed. Although *H. pylori* eradication is advantageous when intestinal metaplasia is identified, follow-up endoscopic examinations should be considered in patients with intestinal metaplasia and in the older age range to detect more progressive conditions [24]. The findings also indicated that precancerous conditions could be found in normal-appearing mucosa. Considering that precancerous conditions may become stomach cancer, detecting them especially in dyspeptic patients older than 40 years with *H. pylori* infection and opium addiction is regarded as a high priority.

*H. pylori* infection rates are very high in different parts of Iran, with a prevalence rate ranging from 69 to 89%, and they do increase with age [25–27]. Population-based studies have reported that > 80% of adults aged ≥40 years are infected and that 64.5% of these infections occurred in dyspeptic patients [13]. Our study revealed a similar high rate (80.2%).

The pathogenesis of gastric cancer is multifactorial and associated with a degree of precancerous conditions [23, 28, 29]. Gastric atrophy, dysplasia, and intestinal metaplasia are considered the main precancerous conditions of the stomach and they develop, although slowly, during continuous *H. pylori* infection [30], that is therefore claimed to be a crucial risk factor of gastric cancer, especially outside the cardia [1]. The presence of precancerous conditions in dyspeptic patients in our study is in line with other studies in Iran but not as common as that reported by studies of Chinese and Asian cases [31–33]; however, it is more frequent than what is reported in western countries [34–36].

Although the gold standard for *H. pylori* detection is gastric biopsy, a study in Mashhad, Iran has shown detection of this infection in 698 out of 814 (85.75%) patients by the urea breath test (UBT) [37], which is just slightly higher than our results. In Ardabil, a high-risk region of gastric cancer in north-western Iran, histological results for *H. pylori* were found positive in almost 90% of the cases examined [14]. As exemplified by a report of 589 positive cases out of 736 (80.0%) examined dyspeptic patients in Mongolia, a country known for its high rates of gastric cancer mortality [38], this infection is indeed common.

The high prevalence of *H. pylori* infections in Iran is an important point due to the overwhelming evidence of a connection between this bacterium and cancer of the stomach. The mechanism involved seems have to do with *H. pylori* inducing over-expression of COX-2, a cyclooxigenase enzyme that catalyses the conversion of arachidonic acid into prostaglandins, higher levels of which have been found in gastric carcinoma and precancerous conditions [39, 40]. COX-2 expression is positively associated with histological status [41–43], and the COX-2/prostaglandin pathway induced by *H. pylori* almost certainly plays an important role in gastric carcinogenesis [44–47]. The drug celecoxib has been shown to inhibit both the flagellar movement and colonization of the *H. pylori* bacterium, and a recent population-based study reveals that treatment with this drug has beneficial effects on advanced gastric conditions [48]. Other studies have shown that the drug can significantly reduce the risk of cancer of the colon, lung, breast and prostate [49–51].

Screening and treatment of *H. pylori* should be encouraged, especially in north-eastern Iran as both *H. pylori* infection and gastric cancer are common in this region [52]. As found in the present study, it is important to consider presence of *H. pylori* infection in the stomach of dyspeptic patients even with normal macro-endoscopic results. That biopsies facilitate the diagnostic approach is further emphasized in a study showing *H. pylori* infection in 50% of normal-appearing gastric mucosa [24], and AGA recommends taking biopsies also of normal-appearing body and antrum of the stomach to explore this potential [15]. In consequence with this strong evidence, we feel that EGD alone is not sufficient for detecting all kinds of *H. pylori*-related inflammatory and precancerous gastric conditions in dyspeptic patients. However, studies on cost versus benefit of regularly adding biopsy studies are needed to complement current findings [53, 54].

The prevalence is affected by many factors, e.g., residency, ethnicity, age, socioeconomic aspects [55, 56], living conditions, lifestyle, hygiene status and industrialization [57–59]. Smoking is an independent risk factor for different types of gastrointestinal cancers and previous studies in Iran have revealed direct links between smoking and cancers [27, 60]. However, this study did not reach anywhere near statistical significance between smoking and precancerous stomach conditions; on the other hand, this could be due to the relatively low number of smokers in the cohort examined. Interestingly, regular use of NSAID was found to be negatively associated with appearance of this kind of conditions and so was aspirin but not at a significant level; again possibly due to the low number of regular aspirin takers in the cohort. A few cohort studies have also reported that NSAIDs consumption is associated with a reduced risk of gastrointestinal cancers, including gastric cancer [61, 62]. However, large-scale, clinical trials are needed to recommend it as protective for gastric cancer. The wider use of the drug celecoxib can also be contemplated, but here we need diagnosis before treatment. Indeed, the widespread use of drugs is fraught with unforeseen side effects and should not be advocated without strong backing evidence.

Two studies in Ardabil, a high-incidence gastric cancer province in Iran, revealed that opium use, water pipe inhalation and high salt intake are risk factors for this cancer [14, 63]. Also in our study, *H. pylori* infection was more common in patients addicted to opium. Further, opium addiction was more common in patients with intestinal metaplasia and dysplasia, although regression analysis revealed no significant association between them. The association between opium use and human cancer have been discussed for a long time with different mechanisms suggested. For example, there is some evidence that opium could increase the ethylation of DNA through reduction of N-nitrosamines and N-nitrosodimethylamine [64]. Also, it has been shown that opiates could work as cancer promoters by damaging human immune function, activating angiogenesis and tumour neovascularisation and also increasing N-nitrosamines and related materials by changing pharmacokinetics [64]. Furthermore, since addicted people often do not keep a personal high hygiene status, *H. pylori* infection may be more common among them. In spite of this collected evidence, a clear cause effect study is needed to confirm this association.

Our study had some limitations. First, Information about proton pump inhibitor (PPI) consumption was not available in this study. Some patients had previous use of PPI and some were frequently self-medicated, so they could not discontinue PPI for an adequate period before endoscopy. Although consistent with previous studies [65, 66] and AGA guidelines [15] we obtained biopsies both from antrum and body for the detection of *H. pylori* infection, but PPI consumption may reduce the colonization density of *H. pylori* and leads to false negative results at the histopathology assessment [67]. Second, the quantitative measurements of opium and alcohol abuse as well as use of NSAIDs and aspirin or other drugs were not addressed in the study. Furthermore, due to the high cost of pathology in our setting, we followed the AGA guidelines [15] and put all the samples together. Thus, it is not possible to classify according to the Sydney system, because in this system, a biopsy should be taken separately from body, antrum, and incisura. However, if precancerous conditions were found in the endoscopy surveillance, separate samples should be taken from different gastric locations. However, although this was not the aim of the current study, this kind of surveillance should be addressed by future studies. These limitations impose a lack of generalizability of the findings, but we still think it will be useful for low-and middle-income countries especially in the Middle East where there is a restriction of the resources available like in Iran.

## Conclusion

This study shows that endoscopic biopsies, even from normal-appearing gastric mucosa, increase the chance of finding gastric cancer at the precancerous stage especially in patients infected with *H. pylori*. There is a well-supported connection between *H. pylori* infection and precancerous and cancerous conditions and as this infection is unusually common in parts of Iran, large-scale national screening may be beneficial. Finding patients at an early stage would save lives and reduce costs.

## Data Availability

The datasets generated and/or analysed during the current study are available in the Harvard Dataverse repository, [10.7910/DVN/RVQZDG].
